# Metabolic and Neuroenergetic Effects of Intranasal Vitamin C Application in the Human Brain

**DOI:** 10.3390/nu17243875

**Published:** 2025-12-11

**Authors:** Lena-Christin Ingwersen, Alina Kistenmacher, Uwe H. Melchert, Kerstin M. Oltmanns

**Affiliations:** Section of Psychoneurobiology, Center of Brain, Behavior and Metabolism, University of Luebeck, 23562 Luebeck, Germany

**Keywords:** ascorbic acid, obesity, cerebral high-energy phosphates, adenosine triphosphate (ATP), phosphocreatine (PCr), neuroprotective, glucose metabolism, insulin resistance

## Abstract

**Background**: Compared with normal weight, obese individuals display a variety of deviant measures in neuroenergetic status, food intake behavior, glucose metabolism, and circulating vitamin C levels. A chronically lowered neuroenergetic content is associated with increased food intake and disturbed glucose metabolism in obesity. In turn, a vitamin C deficiency found in obesity may be connected to these disturbances. Therefore, we investigated the effects of vitamin C application in the human brain. **Methods:** We intranasally applied vitamin C (80 mg ascorbic acid/day) vs. placebo for 8 consecutive days in 15 normal weight (BMI 20–25 kg/m^2^) and 15 obese (BMI > 30 kg/m^2^) men. The neuroenergetic content of adenosine triphosphate (ATP) and phosphocreatine (PCr) was assessed by ^31^phosphorous magnetic resonance spectroscopy, a non-invasive real-time technique to measure high-energy phosphate compounds in living tissues. Peripheral vitamin C, glucose, and insulin concentrations were measured, and spontaneous food intake was quantified by the standardized buffet test. **Results:** In the obese group, vitamin C application acutely suppressed the physiological insulin response on the first experimental day (*p* = 0.003). The following eight days of intranasal vitamin C led to higher serum vitamin C concentrations as compared to placebo (*p* = 0.011), compensated for the missing food intake-induced serum vitamin C rise (*p* ≤ 0.002), and attenuated a PCr decline (*p* = 0.008) in this group. Correlation analyses revealed a general link between serum vitamin C concentrations and the neuroenergetic state in both groups (*p* ≤ 0.033). Food intake was not influenced. **Conclusions:** Intranasal vitamin C application acutely improves insulin sensitivity, compensates for a vitamin C deficiency, and may act in a neuroprotective way in obese men. It could therefore be a future candidate as an adjuvant therapeutic option in obesity treatment.

## 1. Introduction

Appetite, food intake behavior, and systemic energy homeostasis are regulated by the central nervous system in a synchronized fashion, with the current level of high-energy phosphates (i.e., adenosine triphosphate (ATP) and phosphocreatine (PCr)) being considered as key regulators [1]. It has been shown that a lowered content of high-energy phosphates in the brain fosters the intake of high-caloric food and drives appetite [2]. Moreover, body weight and cerebral high-energy phosphate content are known to be negatively related to each other [3]. Various interventions, which elevate the neuroenergetic content—such as i.n. insulin application or transcranial direct current stimulation (tDCS)—lead to reduced appetite, decreased food intake, and enhanced glucose tolerance [2,4,5]. Specifically, the last point is of considerable importance in obesity because those affected mostly exhibit an impaired glucose metabolism or develop diabetes mellitus due to an insufficient effectiveness of insulin despite high serum concentrations, i.e., insulin resistance [6]. Amongst many further alterations found in obesity when compared with normal weight people, one aspect is a vitamin C deficiency [7,8,9]. At first sight, a relationship between neuroenergetic content and vitamin C deficiency appears to be rather far-fetched, but a second look indeed reveals a connection as vitamin C plays an important role in cellular energy synthesis in the human brain [10]. Vitamin C is a vital antioxidant molecule, which serves as a co-factor in several important enzyme reactions and protects mitochondria and thereby energy synthesis of the respiratory chain from oxidative damage [11].

Based on the fundamental function of vitamin C in energy synthesis [10,11] and a previously shown link between Vitamin C and neuroenergetics [12], we assumed that daily i.n. application of vitamin C for 8 consecutive days would affect the neuroenergetic state and—in consequence—also food intake behavior as well as glucose metabolism in obese and normal weight men. Serum vitamin C concentrations were measured to determine whether the known vitamin C deficiency of obese participants could be compensated by i.n. substitution.

## 2. Materials and Methods

### 2.1. Participants

This study examined two different groups: A group of 15 obese male participants (mean body mass index (BMI) 37.0 ± 5.1 kg/m^2^) as well as a normal weight group of 15 healthy male volunteers (mean BMI 22.0 ± 1.6 kg/m^2^), with no difference in age (obese: 25.7 ± 3.1 years vs. normal weight: 23.7 ± 3.4 years, *p* = 0.095, *t*-test). Volunteers with acute or chronic internal, neurological, or psychiatric diseases, diabetes mellitus (also in first-degree relatives), as well as acute or chronic intake of medication or drugs were excluded from the study. Further exclusion criteria were nicotine or alcohol abuse. Participants were informed not to pursue high-performance sports or do any kind of shift work during their participation in the study. Each participant gave written consent. Our study was carried out in accordance with the Declaration of Helsinki (2013) of the World Medical Association. Approval was granted by the ethics committee of the University of Luebeck (registration code: 13-238) on 4 July 2014.

### 2.2. Experimental Design

This study was performed in a randomized, placebo-controlled, cross-over design. Our investigation comprised the daily application of i.n. vitamin C vs. placebo for 8 consecutive days each. Conditions were separated by a minimum of two weeks. On the experimental days 1 and 8, participants arrived at the laboratory at 6:15 a.m. After body weight measurement (Tanita BC-418MA, TANITA EUROPE B.V., Amsterdam, The Netherlands), an intravenous forearm catheter was placed for taking blood samples. To ensure the fasting state of each participant, blood glucose concentrations were initially determined (B-Glucose-Data-Management, HemoCue GmbH, Grossostheim, Germany). After a 20 min rest in a lying position, body composition was determined by Body Impedance Analyzer Nutribox (NutriPlus© 5.4.1. Data Input GmbH, Pöcking, Germany) via application of 0.8 mA 50 kHz with a precision range of ±3%. Subsequently, the first blood sample and a baseline sequence of ^31^phosphorous magnetic resonance spectra (^31^P-MRS) were taken, followed by the application of a nasal spray containing 100 mg/mL vitamin C or 0.9% sodium chloride, as described in the application protocol below. A series of 7 further ^31^P-MR spectra series were taken for about 5 min each. Further blood samples were taken 10 and 35 min after i.n. application. Afterwards, the participants were brought to a different room, where an ad libitum buffet was offered for 30 min. In order to avoid any psychological influence on spontaneous food intake, the subjects were not informed that their food consumption would be quantified. At last, blood samples were taken after ingestion. Participants were released at 8:30 a.m. On day two and for the following seven days, the intervention comprised daily application of the nasal spray at 8:00 a.m. in the laboratory setting.

### 2.3. Intranasal Vitamin C Application Protocol

The nasal spray was applied with a commercial applicator (Aero Pump GmbH, Hochheim am Main, Germany). In a seated position, each participant was instructed to start the application procedure with two to three pumps in the air and to inject the spray first into the right nostril and then the left nostril (0.1 mL each), followed by a rapid and deep inhalation through the nose. This procedure was repeated four times, exactly separated by 30 s. The vitamin C spray contained 100 mg/mL vitamin C (Rotexmedica GmbH, Trittau, Germany), i.e., a total amount of 80 mg of vitamin C was administered each day. The chosen concentration was based on reference values for daily vitamin C intake, which is approximately 110 mg/day for a person of normal weight [13]. Furthermore, the tolerable upper intake level, which is 2000 mg/day for vitamin C [14], was taken into account. A pretest revealed that concentrations above 80 mg of vitamin C caused burning sensations due to an irritated nasal epithelium and thereby would have rendered blinded testing impossible.

The placebo solution consisted of a 0.9% sodium chloride solution (B. Braun Melsungen AG, Melsungen, Germany). Both nasal sprays were administered in an identical way, and the application was guided and controlled by the study instructor in a standardized way.

### 2.4. ^31^P-Magnetic Resonance Spectroscopy Measurements

^31^Phosphorus magnetic resonance spectroscopy (^31^P-MRS) of the brain was conducted in a 3.0-Tesla magnetic resonance scanner (Achieva 3T, Philips Medical Systems, Best, The Netherlands) by using a doubled-tuned ^1^H/^31^P-headcoil (Advanced Imaging Research, Cleveland, OH, USA) before and after the i.n. application of vitamin C or a placebo solution. This non-invasive in vivo method measures the high-energy phosphate content of the brain [15]. Key metabolites are adenosine triphosphate (ATP) and phosphocreatine (PCr) [16]. A repetition time of 4500 ms with a three-dimensional chemical shift imaging sequence (6 × 6 voxel in 3 slices: 24 cm × 24 cm × 12 cm field of view, 6 kHz bandwidth, 1024 data points, 5:00 min measuring time) was used to attain sufficient relaxation of the phosphorus metabolites. Spectral resolution was improved by using ^1^H-decoupling during receiving (wideband alternating-phase technique for zero-residual splitting WALTZ) [17] and nuclear Overhauser effect [18]. Therefore, the second channel of the head coil for transmitting on the ^1^H-resonance frequencies was used. For the calculation of high-energy phosphate levels and to maximize the signal-to-noise ratio, 4 voxels of the second slice, i.e., 8 × 8 × 4 cm^3^ volume of interest (VOI) were selected, which represent the tissue above the corpus callosum containing parts of the motor and sensory cortex, the subcortical area of the gyrus cinguli, and white matter. Standardized VOIs were localized by taking scout images (sagittal, coronal, and transversal) before spectra measurements to ensure comparability of the spectra. Evaluation of the spectra data was carried out by a Java-based graphical user interface for in vivo quantification of MRS signals (jMRUI 4.0) with zero filling to 4096 data points, which were apodized by a 15 Hz Lorentzian filter. Calculation of peak positions and intensities was performed using the Advanced Method for Accurate Robust and Efficient Spectral fitting (AMARES) algorithm [19]. ATP levels were calculated as the sum of α-, β-, and γ-ATP [20,21]. Cerebral high-energy phosphate peak areas are represented as single ATP and PCr. PCr and ATP represent the overall cerebral high-energy phosphate turnover. In a bidirectional manner, ATP is formed by PCr and vice versa by creatine phosphokinase in a molar ratio of 1:1 [20]. ^31^P-MR spectra were measured in 5 min intervals. One spectra series was recorded as a baseline value before the interventions. A further seven spectra series were obtained up to 40 min after the intervention.

### 2.5. Standard Buffet Test

For the ad libitum buffet, participants were taken to a different room. To avoid disruptive factors, the buffet was always arranged in an identical way. Participants were not informed that food consumption would be quantified. Instead, they were told that the buffet was meant as a reward for participating in the study in a fasting state. They were offered 30 min to eat. All food was weighed before and after participants’ food intake. Overall, the entire buffet contained around 6800 kcal from 200 g protein, 325 g fat, and 730 g carbohydrates.

### 2.6. Handling and Analysis of Blood Samples

Blood samples were centrifuged (Sigma 3-16K, Sigma Laborzentrifugen GmbH, Osterode am Harz, Germany) and the supernatants were stored at −80 °C (Thermo Scientific Forma Serie 900 Model 906, Thermo Fisher Scientific Inc., Waltham, MA, USA) until laboratory analyses. For the stability of vitamin C, serum tubes included an additive and the tubes were stored in the dark until further processing. Plasma glucose was measured by the AU 5800 System (Beckman Coulter; inter-assay coefficient of variation (CV) < 0.70%, intra-assay < 1.25%). Serum insulin concentrations were measured by ElectroChemiLuminescence (ECL) immunoassay (Elecsys, cobas e 411 and 602, immunoassay-Systems, Roche Diagnostics, Mannheim, Germany; inter-assay (CV) < 2.0%, intra-assay CV < 2.8%). Serum vitamin C concentrations were measured by high performance liquid chromatography (HPLC) ultraviolet (UV) method (inter-assay CV < 7.7%; intra-assay CV < 5.5%).

### 2.7. Statistical Analyses

Data are presented as mean values ± standard error of the mean (SEM). Statistical analyses were performed by Superior Performing Software System Version 29.0 (SPSS Inc. Chicago, IL, USA) and based on analyses of variance (ANOVA) for repeated measurements. Normal distribution of the data was proven by Shapiro–Wilk test. If necessary, Greenhouse–Geisser correction was applied. Data points exceeding 1.5 times the interquartile range were quantified as outliers and excluded from further analysis. Data, which compared vitamin C with placebo were analyzed according to “treatment” and “time” (time of data collection). ANOVA was calculated for inner-subject factors (within obese and normal weight group) and for between-subject factors (between obese and normal weight group). Accordingly, paired sampled *t*-tests were conducted within, and the unpaired *t*-test was compared between groups. In addition, bivariate correlation analyses were conducted according to Pearson (normal distributed data) or Spearman (nonparametric data). The testing comprised *n* = 15 in each condition and in each group. High-energy phosphate analyses comprised *n* = 14 in the normal weight group due to interruption during ^31^P-MRS measurements in one subject. For body fat mass analyses, on day one *n* = 14 were analyzed in the normal weight group due to technical difficulties with BIA measurements in one subject. A *p*-value < 0.05 was considered significant.

## 3. Results

### 3.1. Serum Vitamin C Concentrations

**Figure 1:**
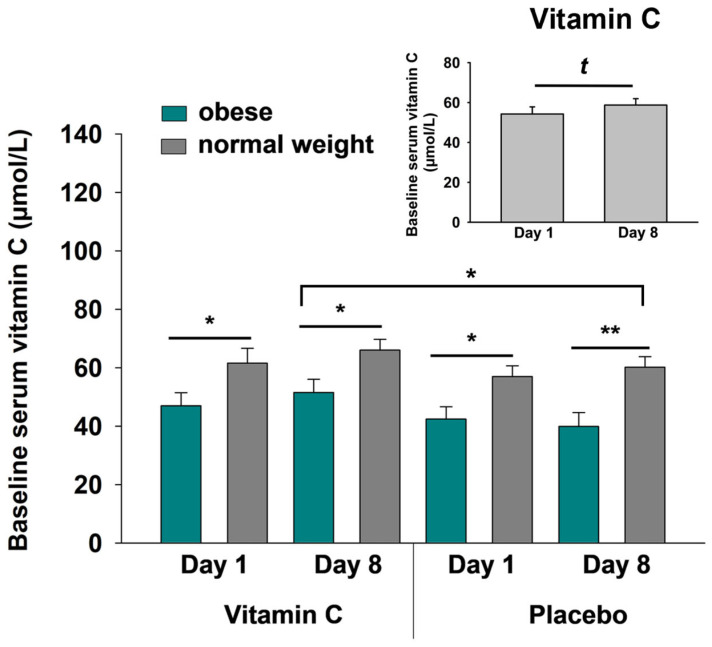
Baseline serum vitamin C concentrations (µmol/L) on day 1 and day 8 prior to i.n. vitamin C or placebo application in obese (green) and normal weight (dark gray) participants. All data are presented as mean values ± SEM; *t*-tests were performed with ^t^ *p* < 0.10, * *p* < 0.05, and ** *p* < 0.01.

Our data confirm previous insight that obese individuals generally display lower serum vitamin C concentrations than normal weight people (*p* ≤ 0.041 for all baseline measurements, *t*-tests; Figure 1). After merging both groups, analyses revealed that the i.n. application of vitamin C resulted in a strong trend towards an increase over time from day 1 to day 8 (*p* = 0.051; Figure 1, small insert). In the obese group, 8 days of i.n. vitamin C substitution led to significantly higher serum vitamin C concentrations as compared to the placebo intervention (*p* = 0.011, *t*-test; Figure 1). This effect was not observed in normal weight participants (*p* = 0.07, *t*-test).

**Figure 2:**
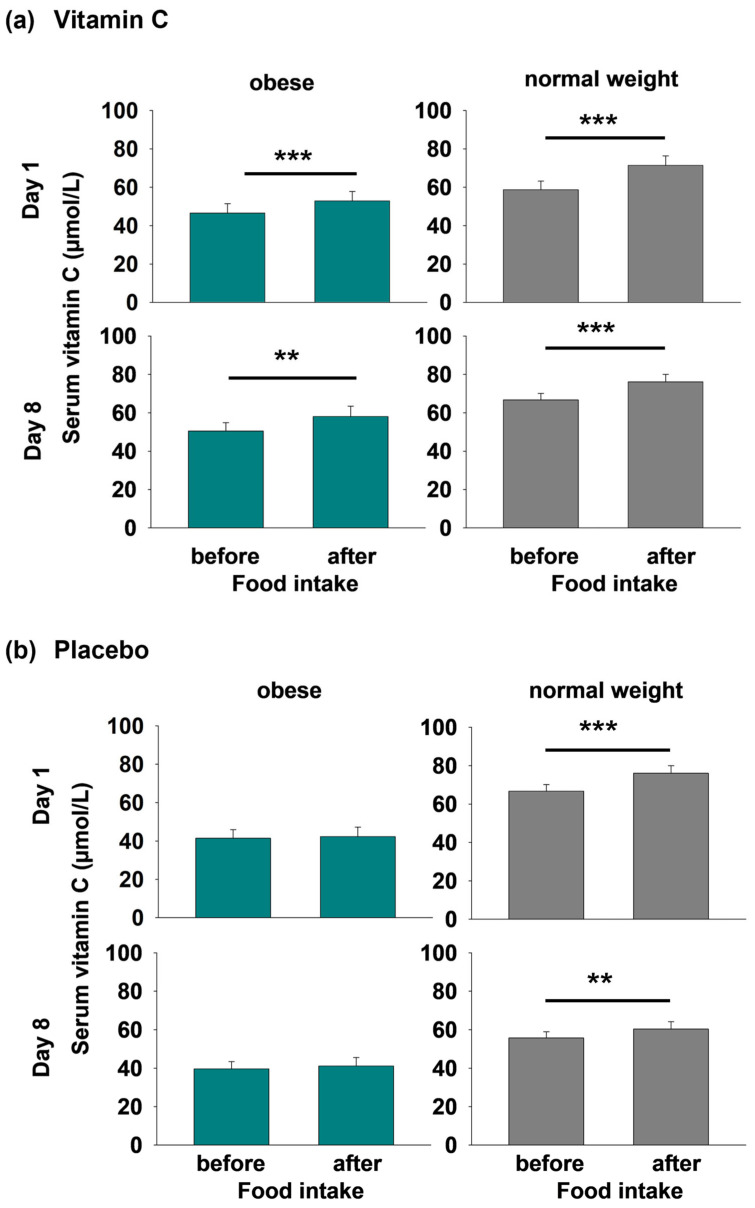
Serum vitamin C concentrations (µmol/L) on day 1 and day 8 in the vitamin C (**a**) and the placebo condition (**b**) before and after food intake in obese (green) and normal weight (gray) participants. All data are presented as mean values ± SEM; *t*-tests were performed with ** *p* < 0.01, and *** *p* < 0.001.

Food intake caused a distinct increase in serum vitamin C concentrations upon i.n. vitamin C application both in the obese and the normal weight group (obese—day 1: *p* < 0.001, day 8: *p* = 0.002; normal weight—day 1: *p* < 0.001, day 8: *p* < 0.001; *t*-tests, Figure 2a). This effect was also observed in the placebo condition in the normal weight group (day 1: *p* < 0.001, day 8: *p* = 0.015, Figure 2b) but was clearly missing in obese participants (day 1: *p* = 0.187, day 8: *p* = 0.273, Figure 2b). In normal weight participants, the rise from pre- to post-prandial serum vitamin C concentration was significantly higher upon i.n. vitamin C administration compared to placebo (day 1: *p* < 0.001, day 8: *p* = 0.002, *t*-test, Figure 2a,b).

### 3.2. Systemic Glucose Metabolism

As expected, comparisons of the obese with the normal weight group revealed significantly higher fasting plasma glucose concentrations on both examination days for the obese participants, regardless of the intervention (*p* ≤ 0.003 for all comparisons). After food intake, there was a comparable increase in glucose concentrations regardless of group and condition (*p* < 0.001 for all comparisons, ANOVA and *t*-tests). No significant effects of vitamin C administration on blood glucose concentration were found (*p* > 0.05 for all comparisons).

**Figure 3:**
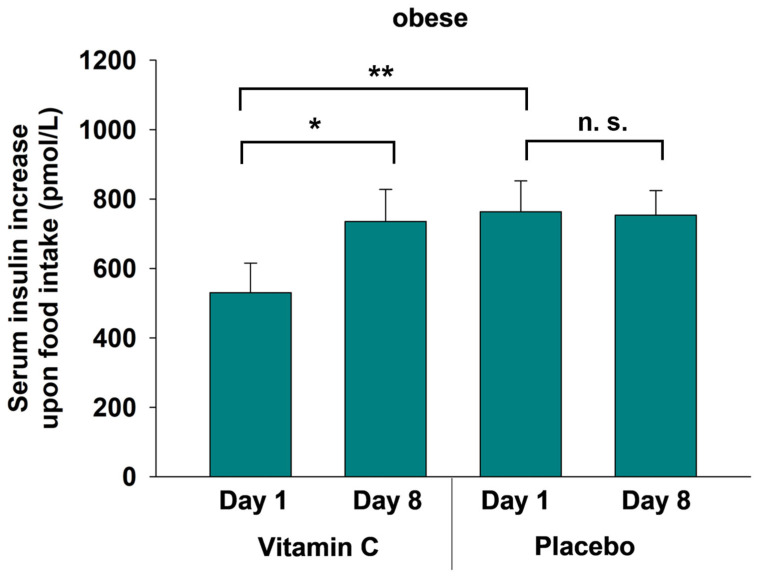
Increase in serum insulin concentrations (pmol/L) upon food intake on day 1 and day 8 in the vitamin C and placebo conditions in the obese group. All data are presented as mean values ± SEM; *t*-tests were performed with * *p* < 0.05, ** *p* < 0.01 and n.s. = not significant.

Similarly, fasting serum insulin concentrations were significantly higher in the obese compared to the normal weight group, regardless of time and conditions (*p* < 0.001, for all group comparisons, *t*-tests). As with glucose concentrations, there was a temporal increase in insulin concentrations due to food intake, which was independent of group and condition (*p* ≤ 0.004 for all comparisons). In the normal weight group, the insulin increase was similar in both conditions (*p* ≥ 0.814). However, in the obese group, we observed a distinctly lower insulin response upon food intake after vitamin C application as compared with the placebo on the first day of measurements (*p* = 0.003, *t*-test, Figure 3). This suppressed insulin secretion in the vitamin C condition is apparently reversible with time as on day 8, the response to food intake was significantly higher than on day 1 (*p* < 0.017) and comparable to the placebo condition (*p* = 0.412, Figure 3). This transient insulin-suppressive effect of vitamin C was not found among normal weight participants (*p* = 0.814).

### 3.3. Cerebral High-Energy Phosphates

**Figure 4:**
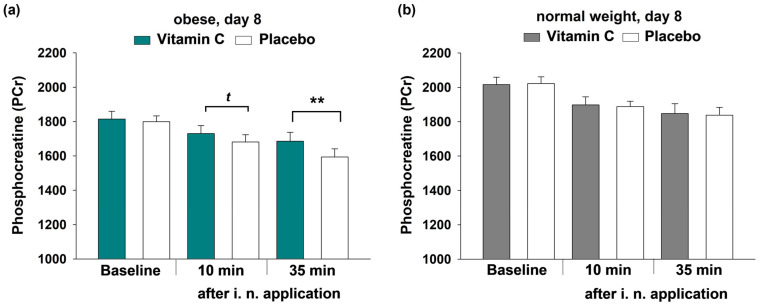
Cerebral Phosphocreatine (PCr) content of the obese (**a**) and the normal weight group (**b**) on day 8 before and after i.n. vitamin C application (10 and 35 min) vs. placebo. All data are presented as mean values ± SEM; *t*-tests were performed with *^t^ p* < 0.1 and ** *p* < 0.01, values were determined by calculating the area under the spectral peak, therefore no units are provided.

Confirming the previous insight, comparisons of the obese with the normal weight group revealed significantly lower cerebral ATP and PCr levels in both conditions and on both examination days in obese participants (*p* ≤ 0.009 for all comparisons). Over the course of the experimental day, ATP and PCr contents decreased in a comparable way regardless of group and condition (ANOVA time for all comparisons: *p* ≤ 0.006). While we did not find any significant effects of vitamin C application in terms of the cerebral ATP content (*p* ≥ 0.366 for all comparisons), PCr levels were apparently influenced by vitamin C. Eight days of vitamin C treatment attenuated the PCr drop by trend 10 min after i.n. application and this trend became significant after further 15 min (10 min: *p* = 0.082, 35 min: *p* = 0.008, Figure 4a). In contrast, normal weight participants did not show this effect of vitamin C application (*p* ≥ 0.522 for all comparisons, Figure 4b).

**Figure 5:**
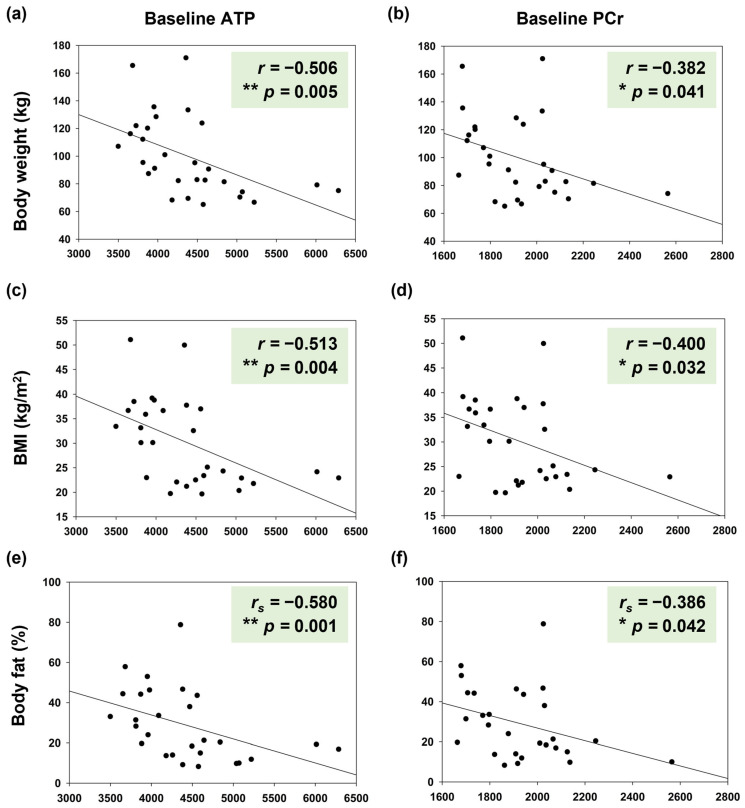
Correlation analyses of body weight (kg; (**a**,**b**)), BMI (kg/m^2^; (**c**,**d**)), and relative body fat mass (%; (**e**,**f**)) with baseline ATP and PCr levels in the brain (data shown for vitamin C application on day 1, merged data of both groups). *r*: Pearson correlation coefficient, *r_s_*: Spearman correlation coefficient, *p*: *p*-value, * *p* < 0.05 and ** *p* < 0.01; ATP: adenosine triphosphate, PCr: phosphocreatine.

Merging both participant groups, analyses confirmed the known negative correlation of body weight with cerebral baseline ATP and PCr content (*r* ≤ −0.382, *p* ≤ 0.041 for all correlations, Figure 5a,b). In analogy, there was a negative correlation between BMI and cerebral baseline ATP and PCr (*r* ≤ −0.400, *p* ≤ 0.032 for all correlations, Figure 5c,d). Adding new insight into the well-known relationship between body mass and cerebral high-energy phosphate content, we discovered that this connection is seemingly based on body fat content as we found significant negative correlations between brain ATP as well as PCr levels and percent of body fat, i.e., the higher the body fat content the lower the neuroenergetic level in participants (*r_s_* ≤ −0.386, *p* ≤ 0.042 for all correlations, Figure 5e,f).

### 3.4. Correlation of Serum Vitamin C and Cerebral High-Energy Phosphates

**Figure 6:**
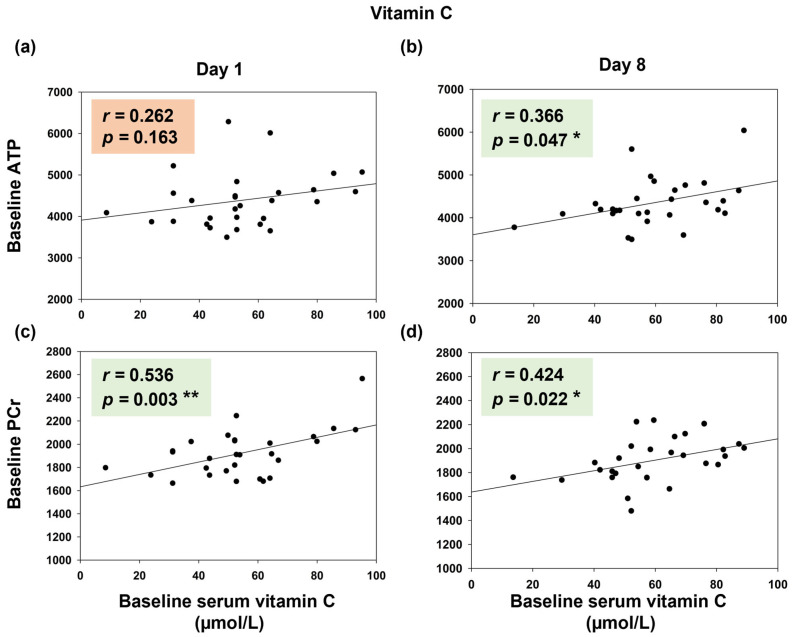
Correlation analyses of baseline serum vitamin C concentrations (µmol/L) with baseline cerebral ATP (**a**,**b**) and PCr content (**c**,**d**) on day 1 and 8 (vitamin C condition, both groups merged). *r:* Pearson correlation coefficient, *p*: *p*-value, * *p* < 0.05 and ** *p* < 0.01; ATP: adenosine triphosphate, PCr: phosphocreatine.

Merging both groups, our correlation analyses revealed a significant positive correlation between baseline serum vitamin C concentrations and baseline ATP content prior to vitamin C application on day 8 (*r* = 0.366; *p* = 0.047, Figure 6b), whereas there was no significant correlation on day 1 (*r* = 0.262; *p* = 0.163; Figure 6a). Similarly, we found a significant correlation between baseline PCr levels and baseline serum vitamin C concentrations in the vitamin C condition on both examination days (day 1: *r* = 0.536, *p* = 0.003, day 8: *r* = 0.424, *p* = 0.022, Figure 6c,d). In the placebo condition, no correlations were found.

### 3.5. Food Intake

The obese group generally tended to consume more food than normal weight participants (vitamin C—day 1: *p* = 0.098, day 8: *p* = 0.063; placebo—day 1: *p* = 0.094). Only on day 8 of the placebo intervention, this trend was not evident (*p* = 0.286). The trend towards higher amounts of consumed food in the obese group was apparently based on higher protein intake than in normal weight individuals (*p* ≤ 0.049 for all comparisons). Vitamin C application did not have any influence on food intake as compared to placebo neither by time nor by group (obese—day 1: *p* = 0.140, day 8: *p* = 0.402; normal weight—day 1: *p* = 0.376, day 8: *p* = 0.789). Notwithstanding, on day 1 of the examination there was a trend towards reduced food intake upon vitamin C application when merging the obese and the normal weight group (*p* = 0.087).

## 4. Discussion

### 4.1. Serum Vitamin C

Our data confirm previous observations that overweight is related to lowered vitamin C concentrations as compared to normal weight people [7]. To overcome this deficiency, obese people require extra vitamin C dosages of at least 50 µmol/L to reach normal concentrations in the blood. The vitamin C concentration plateau (steady-state) in healthy humans lies at 60–80 µmol/L [9]. Although i.n. application should rather exert an indirect effect on circulating vitamin C concentrations, we found that obese subjects may benefit from it, as our data show significantly higher serum vitamin C concentrations after 8 days of i.n. vitamin application as compared with placebo treatment. This effect could be due to the fact that absorption or breakdown of vitamin C may be disturbed in obese individuals, leading to reduced vitamin C concentrations. Therefore, intranasal vitamin C application alleviates the vitamin C deficiency in obese individuals. The intranasal way of compensating vitamin C deficiency may not only help to increase circulating vitamin C concentrations in the periphery but also improve the oxidative status in the brain and thereby exert some neuroprotective effects, particularly in overweight individuals.

Another result of our study showed that not only in the obese but also in the normal weight group, the physiological increase in vitamin C concentrations upon food intake could be further increased by preceding i.n. vitamin C administration. Previous work describes that the oral uptake of vitamin C leads to a measurable rise in vitamin C levels in the blood, with the peak occurring two to three hours after oral administration. The bioavailability of vitamin C in synthetic form or in the presence of food or fruit juices is comparable [22]. In our study, we detected an increase in vitamin C concentrations in the circulating blood already 30 min after orally consuming vitamin C (approximately 68 mg, which corresponds to approximately 200 mL of orange juice in our standard buffet) [23,24]. It therefore seems likely that the increase in serum vitamin C levels observed in our study was caused by vitamin C uptake from consumed foods and that i.n. vitamin C application may have boosted the absorption of vitamin C from food.

Notwithstanding, we observed that the physiological vitamin C rise upon food intake was missing in obese participants unless i.n. application occurred. This finding leads to speculate that overweight people may have a hampered vitamin C absorption from food. I.n. vitamin C administration in obese individuals may potentially improve the oxidative status in the brain, thereby initiating processes that support the absorption of vitamin C from food.

### 4.2. Glucose Metabolism and Vitamin C

As expected, plasma glucose and serum insulin concentrations were higher in overweight than in normal weight people, and food intake inherently led to an increase in both parameters in the circulating blood. As is known, high blood glucose concentrations stimulate insulin secretion. Unfortunately, in obesity, this reinforced pancreatic insulin secretion cannot stabilize plasma glucose concentrations due to increasing loss of efficiency, i.e., obese individuals develop insulin resistance [6]. On day one, after administering vitamin C to our obese participants, we observed that despite high blood glucose concentrations, the insulin response after food intake was not expectedly high but, right to the contrary, at the level of normal weight individuals. As plasma glucose concentrations remained stable, this finding indicates an increased insulin efficiency. This result is in line with data from previous work, which demonstrated that vitamin C—administered intravenously—improves insulin efficiency in healthy older adults and diabetes patients [25,26], probably by lowering reactive oxygen species (ROS) levels [27]. However, previous studies mostly used a combination of vitamin C with other supplements, which renders it difficult to specify isolated vitamin C-induced effects and compare them to our findings.

### 4.3. Vitamin C and Cerebral Energy Content

Our data replicate our previous insight that obese people display reduced levels of high-energy phosphates (ATP and PCr) in the brain. This relationship between BMI and neuroenergetic state is not new [3]. However, our current data shed new light on the underlying factor of this relationship: correlation analysis reveals that there is a strong connection between high body fat content and decreased high-energy phosphate levels in the brain. This correlation, therefore, appears to be the result of being overweight and not—as one might assume based on BMI/weight measurements—increased muscle mass.

Beyond this new insight about previous knowledge, we found another interesting neuroenergetic effect in our study: a stable decline in high-energy phosphates (ATP and PCr), which was observed in all participants over the duration of an experimental day. This observation suggests a circadian rhythmicity. Looking at the literature, a circadian rhythm has previously been described for extracellular ATP as quantified in the suprachiasmatic nucleus (SCN), the cortex, and the anterior hypothalamic area (AHA) of rats [28]. Although the found peak in ATP levels varies, most studies show a peak between 6 p.m. and 10 p.m. Regarding PCr and a circadian rhythm, there are no effects described so far. However, i.n. vitamin C application exerted an interesting effect on the neuroenergetic drop in the obese participants of our study. The decrease in PCr levels on day 8 in the obese group was significantly attenuated through vitamin C application as compared with placebo. It would be of interest to look at a later day time to see whether the reduction in PCr persists in obese individuals and possibly influences food intake. In the normal weight group, no such effect was observed. The drop in ATP levels was not influenced by i.n. vitamin C application. In this context, one must consider that we conducted our measurements for a limited period of 2 h in the morning. It is known that the vitamin C content in the brain is considerably higher than in the periphery [29], so it is possible that a longer application period would be required to achieve further effects and maybe also ATP could be affected. Since our study did not collect data over a 24 h period, it is difficult to predict which values would have been measured in the evening. Thinking about the physiological reasons for the hampered neuroenergetic drop upon vitamin C application in obese individuals, we can only speculate. Maybe i.n. vitamin C application prevents a further drop in already lowered PCr content of obese subjects in a neuroprotective way.

In this context, we found further solid correlations between serum vitamin C concentrations and cerebral high-energy phosphate levels. This is in line with previous data, which had described this relationship under conditions of experimentally induced hyperglycemia [12]. In turn, this is compatible with the previously described role of vitamin C in protecting cellular energy synthesis by reducing superoxide and other free radicals, which are a major diffusible byproduct of rapid neuronal mitochondrial metabolism and lipid peroxidation [10]. The addition of vitamin C to cultured cells, brain slices, and brain microsomes prevented lipid peroxidation induced by various oxidizing agents. However, it is difficult to transfer these findings to in vivo conditions, as the oxidant stress induced in these models may have little physiological relevance.

### 4.4. Food Intake

Against our hypothesis, apart from the trend of a higher overall intake due to a higher protein intake in the obese compared to the normal weight group, there were no vitamin C-mediated effects on food intake. However, since the effects on PCr first appeared after 8 days of intranasal vitamin C application, it would be of interest to investigate whether food intake changes after a longer period of intranasal vitamin C application. It would also be relevant to examine whether i.n. vitamin C application has an effect on food intake later in the day and whether this effect persists throughout the day.

Finally, in terms of interpreting our data, a few limiting aspects must be mentioned. It should be noted that only men were examined in this study. Our data may have varied with the incorporation of women in this study, as antioxidants such as vitamin C play an important role in female reproduction and the menstrual cycle, which can lead to variations in vitamin C requirements [30]. Specifically, hormonal fluctuations during the menstrual cycle may increase insulin sensitivity [31] and food cravings in women with premenstrual syndrome [32]. Moreover, the dosage of i.n. vitamin C and the length of the application protocol were chosen rather arbitrarily, as we had no orientation from previous work in this regard. The application period seems to be a very short time interval for detecting measurable metabolic or neuroenergetic changes; therefore, alternative and longer time intervals should be considered. On the other hand, it is also possible that the intranasal administration of vitamin C had only transient effects rather than long-lasting ones, as our effects on insulin show. Also, it would be of interest to know what general use i.n. vitamin C application may have, and what is actually induced within the brain after i.n. vitamin C administration. However, limitations such as the above-mentioned hinder a reliable interpretation. To further clarify this, additional experiments, e.g., in animal models and a follow-up study, would be necessary.

Overall, despite open questions in terms of the underlying mechanisms, vitamin C supplementation exerts some beneficial effects specifically in obesity-caused disturbances. Oral application of vitamin C improves the effectiveness of an energy-restricted diet for reducing body weight [33], adds to reduce oxidized lipoproteins in high- and normal weight, and improves insulin sensitivity in overweight persons [34]. As vitamin C is a strong acid, which can damage the gastric mucosa when taken orally, i.n. administration offers a clear advantage in improving oxidative levels directly in the brain while avoiding damage to the stomach.

## 5. Conclusions

Intranasal administration of vitamin C acutely improves insulin sensitivity, compensates for vitamin C deficiency, and may have a neuroprotective effect in overweight men. It could therefore be considered as an adjuvant therapy option in the treatment of obesity in the future, as it has the advantage of improving oxidative levels directly in the brain.

## Data Availability

The datasets used during the current study are available from the corresponding author on reasonable request.

## Abbreviations

The following abbreviations are used in this manuscript:

^31^P-MRS	^31^phosphorous magnetic resonance spectra
ANOVA	analyses of variance
ATP	adenosine triphosphate
BMI	body mass index
i.n.	intranasal
PCr	phosphocreatine
SEM	standard error of the mean
